# Novel Therapeutic Combination Targets the Growth of Letrozole-Resistant Breast Cancer through Decreased Cyclin B1

**DOI:** 10.3390/nu15071632

**Published:** 2023-03-28

**Authors:** Jankiben R. Patel, Bipika Banjara, Afia Ohemeng, A. Michael Davidson, Stephen M. Boué, Matthew E. Burow, Syreeta L. Tilghman

**Affiliations:** 1 Division of Basic Sciences, College of Pharmacy and Pharmaceutical Sciences, Florida A&M University, Tallahassee, FL 32307, USA; 2 Southern Regional Research Center, United States Department of Agriculture, Agricultural Research Service, 1100 Robert E. Lee Blvd., New Orleans, LA 70124, USA; 3 Section of Hematology and Medical Oncology, School of Medicine, Tulane University, New Orleans, LA 70112, USA

**Keywords:** breast cancer, endocrine resistance, letrozole, aromatase inhibitors, phytochemicals, cancer stem cells, cell cycle, mammospheres

## Abstract

As breast cancer cells transition from letrozole-sensitive to letrozole-resistant, they over-express epidermal growth factor receptor (EGFR), mitogen-activated protein kinase (MAPK), and human epidermal growth factor receptor 2 (HER2) while acquiring enhanced motility and epithelial-to-mesenchymal transition (EMT)-like characteristics that are attenuated and reversed by glyceollin treatment, respectively. Interestingly, glyceollin inhibits the proliferation and tumor progression of triple-negative breast cancer (TNBC) and estrogen-independent breast cancer cells; however, it is unlikely that a single phytochemical would effectively target aromatase-inhibitor (AI)-resistant metastatic breast cancer in the clinical setting. Since our previous report indicated that the combination of lapatinib and glyceollin induced apoptosis in hormone-dependent AI-resistant breast cancer cells, we hypothesized that combination therapy would also be beneficial for hormone independent letrozole-resistant breast cancer cells (LTLT-Ca) compared to AI-sensitive breast cancer cells (AC-1) by decreasing the expression of proteins associated with proliferation and cell cycle progression. While glyceollin + lapatinib treatment caused comparable inhibitory effects on the proliferation and migration in both cell lines, combination treatment selectively induced S and G2/M phase cell cycle arrest of the LTLT-Ca cells, which was mediated by decreased cyclin B1. This phenomenon may represent a unique opportunity to design novel combinatorial therapeutic approaches to target hormone-refractory breast tumors.

## 1. Introduction

Postmenopausal women diagnosed with estrogen-receptor-positive (ER+) breast cancer are candidates for treatment with aromatase inhibitors (AI) such as letrozole or antiestrogens such as tamoxifen or fulvestrant. In many cases, AIs are considered first-line therapy in this setting. Although letrozole is initially effective and reduces tumor burden, some patients develop AI resistance which can progress to metastatic disease resulting in morbidity [1,2]. Once the tumors become resistant to AIs, they exhibit increased growth factor signaling (i.e., human epidermal growth factor receptor 2 (HER2)) [3,4,5], increased cellular motility, and the epithelial-to-mesenchymal transition (EMT) [6]. Unfortunately, these tumors metastasize and aside from chemotherapy, there are limited targeted therapeutic options.

As many patients succumb to metastatic breast cancers, it is increasingly critically to develop strategies to target aberrant pathways that contribute to this pathology. To this end, it is known that cyclin dependent kinase 4 (cdk4) activates the E2F transcription factor which is essential for cell cycle progression. Recent findings demonstrate that ERα-dependent E2F transcription can mediate resistance to estrogen deprivation in human breast cancer [7]. This finding along with others supported the development of cyclin D1-cdk4/6 inhibitors. Currently, for women diagnosed with HER2-negative, hormone-receptor-positive advanced disease, cdk4/6 inhibitors are indicated with endocrine therapy as first-line or second-line treatment [8] and have shown significantly improved efficacy outcomes [9]. Unfortunately, cdk4/6 inhibitors are also associated with resistance that can occur through the loss of the retinoblastoma protein (Rb) or phosphatase and tensin homology (PTEN) [10]; however, some AI-resistant tumors are HER2-positive and are not candidates for cdk4/6 inhibitor treatment.

To date, few studies investigate targeted approaches for AI-resistant breast cancer such as the use of lapatinib [5,11], intermittent letrozole treatment [12,13], histone deacetylase (HDAC) inhibitors [12,14], cdk4/6 inhibitors [15], or co-targeting cdk2 and cdk4/6 [16]. While co-targeting cdk2 and cdk4/6 with endocrine therapy is promising in the pre-clinical setting, most of these approaches are associated with continued tumor proliferation and tumor relapse, and in most cases, failure to respond to treatment can be fatal. As a result, understanding the mechanism(s) of resistance will aid in the identification of potential biomarkers and the development of rational combination targeted approaches.

Soy-containing compounds are gaining interest as potential breast cancer agents [17]. Incidentally, reports indicate that consuming high amounts of polyphenolic phytochemicals are chemopreventive [18]. Glyceollins are members of the flavonoid family of soy-derived phytochemicals, and our group as well as others have experimental evidence implicating its use in cancer prevention and as well as a potential treatment for mammary tumorigenesis [17,19,20,21,22]. Glyceollins are phytoalexins that are composed of three isomers (glyceollin I, glyceollin II, and glyceollin III) that are isolated from activated soy. Previously reports indicate that glyceollin is a novel antiestrogen that binds to the ER and reduces estrogen-induced tumor progression [9]. When letrozole-resistant cells were treated with glyceollin I, cell proliferation and cell motility were attenuated and the EMT was reversed [23]. Although glyceollins as monotherapy are promising in the endocrine-refractory preclinical setting [11,23,24], it is unlikely that monotherapy could significantly decrease tumorigenesis and metastasis in the clinical setting. Therefore, it is plausible that combinatorial therapeutic approaches may be more efficacious.

Given that HER2+ endocrine-resistant tumors are not candidates for cdk4/6 inhibitor therapy, we previously studied the combination of glyceollin and lapatinib in an ER+, aromatase expressing, letrozole-resistant T47D cell line (T47DaromLR) that overexpressed HER2 and mitogen-activated protein kinase (MAPK). In this setting, where the AI-resistant cells remained hormone dependent, combination therapy inhibited proliferation and triggered apoptosis through induction of caspase 7. However, breast tumors are highly heterogenous, and do not all remain hormone dependent as they acquire resistance to endocrine therapy and ultimately may not respond favorably to the same treatments. Therefore, in the current study we treated an EGFR/HER2-positive, hormone-independent AI-resistant breast cancer cell line with glyceollin + lapatinib to explore whether a more aggressive breast cancer subtype would be sensitive to combination therapy.

## 2. Materials and Methods

### 2.1. Cell Culture

We utilized two previously developed breast cancer cell lines. Dr. Angela Brodie kindly provided the AC-1 human breast cancer cells (MCF-7 cells stably transfected with the human aromatase gene) and the long-term letrozole-treated cells (LTLT-Ca). The AC-1 cells were cultured in Dulbecco’s Modified Eagle media (DMEM) (Invitrogen, Carlsbad, CA, USA) supplemented with 5% fetal bovine serum (FBS), 1% penicillin/streptomycin, 1% antimycotic/antibiotic (10,000 U/mL penicillin G sodium; 10,000 mg/mL streptomycin sulfate), and 7.5 µg/mL geneticin (Invitrogen). The LTLT-Ca cells which were previously described [25] mimic resistance to the aromatase inhibitor letrozole. They were cultured in phenol red-free IMEM media (Invitrogen) supplemented with 10% charcoal-stripped fetal bovine serum (CS-FBS), 1% penicillin/streptomycin, 1% antimycotic/antibiotic (10,000 U/mL penicillin G sodium; 10,000 mg/mL streptomycin sulfate), 7.5 µg/mL geneticin (Invitrogen), and 1 µM letrozole (Sigma Aldrich, St. Louis, MO, USA). The cells were maintained in a tissue culture incubator in a humidified atmosphere of 5% CO_2_ and 95% air at 37 °C.

### 2.2. Viability Assays

Proliferation assays were performed as previously described [25]. Briefly, the AC-1 (AI-sensitive) and LTLT-Ca (AI-resistant) cells were seeded at a density of 1 × 10^3^ cells/per well in a total volume of 100 μL. To determine background levels, blank samples were prepared and contained only media. The next day, the cells were treated with control (DMSO), 25 nM androstenedione (Δ4A), 100 nM fulvestrant (ICI), 5 μM lapatinib [15], 10 μM glyceollin (Gly), 5 μM Lap plus 10 μM Gly to determine the effects of the various treatments on both cell lines. Afterwards, resazurin dye (Sigma Aldrich, St. Louis, MO, USA) was added to each well and incubated for 4 h at 5% CO_2_ at 37 °C. Samples were agitated for 1 min and fluorescence was measured at 24, 48, and 72 h using a Biotek Synergy H1 microplate reader (BioTek Instruments, Inc., Winooski, VT, USA) to measure fluorescence intensity at 550 nm excitation/590 nm emission-background wavelengths. All experiments were performed with an *n* ≥ 3 and a total of 3 biological replicates were performed. The proliferative activity was calculated as follows: Proliferative activity = [Fluorescence of viable cells − Fluorescence of blank (media only)].

### 2.3. Wound Healing Assay

The wound healing assay is used as a surrogate marker for cell motility. To create the wound, an Ibidi 2-well culture-insert (Ibidi, Gräfelfing, Germany) was inserted in a 6-well culture plate. Afterwards, 1.5 × 10^5^ AC-1 and LTLT-Ca cells were seeded to create a confluent monolayer. On the following day, the insert was removed causing an area that was void of cells. Serum-free medium was added, and the wound was photographed (i.e., this was considered the initial 0 time point) using the 5× objective on a Zeiss AX10 fluorescence microscope. The cells were treated with control (DMSO), 25 nM Δ4A, 5 μM Lap, 10 μM Gly, 5 μM Lap plus 10 μM Gly and allowed to migrate for 48 h. The wounds were photographed and measured using the Olympus CellSens Standard 1.16 software. The images were compared and analyzed as previously described [25].

### 2.4. Mammosphere Formation Assay

Both cell lines were cultured in standard growth media. Once the cells were 80–90% confluent, they were rinsed with Hank’s Balanced Salt Solution (HBSS) (StemCell Technologies, Vancouver, BC, Canada), gently scraped, and resuspended in MammoCult™ media (StemCell Technologies, Vancouver, BC, Canada). Next, the cells were collected, the supernatant was removed, and the pellet was resuspended MammoCult™ media. Afterwards, 1 × 10^5^ cells were seeded in ultra-low adhesion dishes (CellTreat Scientific Products, Pepperell, MA, USA) and incubated for approximately 7 days and treated with either DMSO, 5 µM Lap, 10 µM Gly, or 5 μM Lap plus 10 μM Gly on Day 5. On day 7, the mammospheres were counted and analyzed using Graph Pad Prism (two-way ANOVA).

### 2.5. Cell Cycle Analysis

Approximately 1.5 × 10^6^ cells were seeded and treated with either (DMSO), 5 μM Lap, 10 μM Gly, and 5 μM Lap plus 10 μM Gly for 48 h. The drug treated media was discarded, the cells were scraped, collected, and the supernatant was removed. The cells were fixed with ice-cold 70% *v*/*v* absolute ethanol, stored at 4 °C overnight, stained with propidium iodide solution, and incubated for 1 h at 37 °C. The stained cells were analyzed for DNA content using a BD FACS Caliber flow cytometer (BD Biosciences, San Jose, CA, USA). The cell cycle phase distribution (G0/G1, S, and G2/M phases) was calculated using Cell Quest Pro software, Version 5.1 and measured using ModFit LT 3.2.1 software (Verity software house Inc., Topsham, ME, USA).

### 2.6. Western Blot Analysis

AC-1 and LTLT-Ca cells were cultured in standard growth media. When the cells were 70–80% confluent, they were treated with vehicle control (DMSO), 5 μM Lap, 10 μM Gly, and 5 μM Lap plus 10 μM Gly for 24 h. The cells were scraped and homogenized in cold RIPA buffer supplemented with a protease inhibitor (Thermo Fisher Scientific, Inc., Waltham, MA, USA). Protein separation, membrane blotting, and antibody probing were conducted as previously described [26] using the following primary antibodies against cyclin B1 (Rb IgG, D5C10) (1:1000), cdk2 (Rabbit mAb, 78B2) (1:1000), p21 (Rabbit mAb, 78B2) (1:1000), p27 (Rabbit mAb, 78B2) (1:1000), β actin (Rabbit mAb, 13E5) (1:1000), or GAPDH (Rb mAb (14C10) (1:1000) (Cell Signaling Technology, Danvers, MA, USA), or against cdk1 (Rabbit mAb, YE324) (1:2000) (Abcam Waltham, Boston, MA, USA). Afterwards, they were then washed and incubated with anti-rabbit secondary antibody. The bands were detected using the Clarity Max Western ECL substrate and visualized using the ChemiDoc XRS imaging system (Bio-Rad, Hercules, CA, USA). The band density was analyzed by Image Lab software (Bio-Rad) and normalized against the housekeeping protein bands (GAPDH or β actin).

## 3. Results

### 3.1. Increased Growth Factor Expression in Hormone-Independent Aromatase-Inhibitor-Resistant Breast Cancer Cells

**Figure 1:**
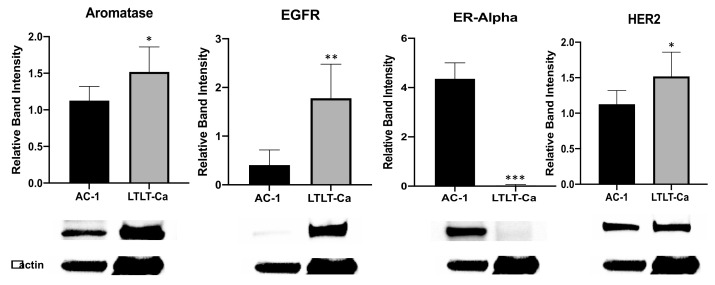
Comparative protein expression profile of letrozole-sensitive cells (AC-1) versus letrozole-resistant cells (LTLT-Ca). The AC-1 and LTLT-Ca cells were assayed by immunoblot with antibodies directed against aromatase, EGFR, ERα, or HER2. β actin was measured as an internal loading control. Quantitative analysis represents the band intensity of the indicated proteins after normalizing to β actin loading control. One-way ANOVA analyses were performed, and AC-1 cells were compared to LTLT-Ca cells. Results are expressed as the mean relative band intensity ± SD (*** *p* < 0.001, ** *p* < 0.01, * *p* < 0.05), and data are representative from one of at least three independent experiments.

Previous reports from our lab and others demonstrate that as cells acquire resistance to letrozole, they lose their dependence on estrogen for growth and utilize growth factor signaling cascades for survival [4,6]. Here, immunoblots were conducted to measure the impact of long-term letrozole resistance on the expression of ERα, aromatase, EGFR, and HER2 (Figure 1). Letrozole-sensitive AC-1 cells were compared to letrozole-resistant LTLT-Ca cells, and the levels of aromatase, EGFR, and HER2 were increased in the LTLT-Ca cells, while ERα was decreased.

**Figure 2:**
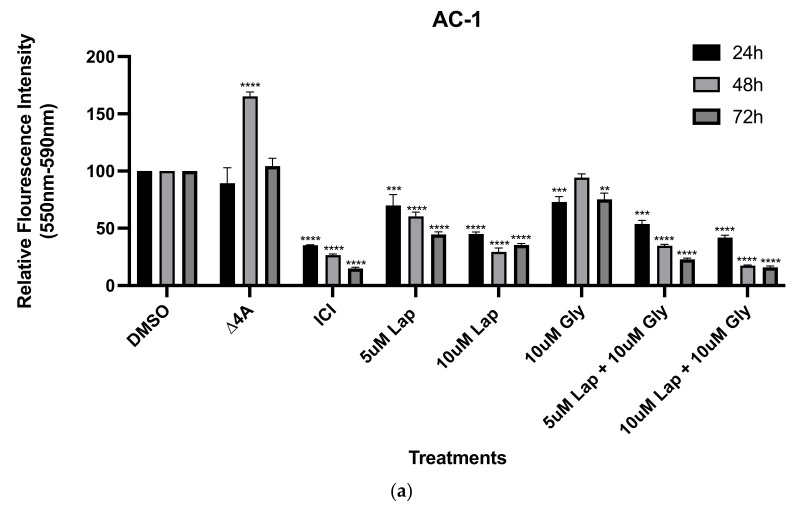

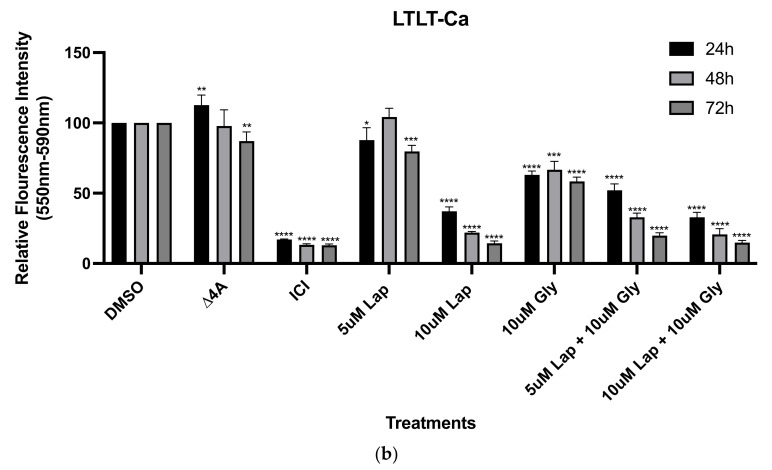
Lapatinib and glyceollin inhibit the proliferation of letrozole-sensitive and letrozole-resistant breast cancer cells. (**a**) The AC-1 cells were cultured in standard growth media and treated with DMSO vehicle control, 25 nM androstenedione (Δ4A), 100 nM ICI (fulvestrant), 10 μM glyceollin, and 5 μM or 10 μM lapatinib ± 10 μM glyceollin, and cell proliferation assays were performed after 24, 48, or 72 h. (**b**) The LTLT-Ca cells were treated as indicated above. The resazurin assay was conducted and the graphs indicate the fluorescent intensity of cells read at 450/590 nM wavelengths after the indicated time points. Two-way ANOVA analysis were performed, and treatments were compared to the DMSO control. Results are expressed as the mean unit ± SD (**** *p* < 0.0001, *** *p* < 0.001, ** *p* < 0.01, * *p* < 0.05) of three independent experiments in triplicate.

Given that the resistant cells were associated with an enhanced growth factor signaling signature and increased aromatase expression, we were interested in determining how proliferation would be impacted by treating the cells with an anti-estrogen (i.e., fulvestrant), the dual HER2 and EGFR inhibitor (i.e., lapatinib), as well as androstenedione (i.e., the substrate for the aromatase enzyme) for 24, 48, and 72 h. We also chose to treat both cell lines with 10 µM glyceollin ± lapatinib, since previous studies from our lab indicated that glyceollin inhibited the growth of estrogen-independent LTLT-Ca cells [23] and combination therapy inhibited the growth of estrogen-dependent T47DaromLR cells [11]. Stimulating the AC-1 cells with androstenedione dramatically increased cellular proliferation while only modest effects were observed in the LTLT-Ca cells (Figure 2). Both cell lines were sensitive to the inhibitory effects of fulvestrant, lapatinib, glyceollin, and glyceollin + lapatinib. Interestingly, although the LTLT-Ca cells expressed higher levels of EGFR and HER2, both cell lines responded similarly to 10 µM lapatinib treatment.

### 3.2. Glyceollin and Lapatinib Inhibit the Migratory Behavior of Letrozole-Sensitive and Letrozole-Resistant Breast Cancer Cells

**Figure 3:**
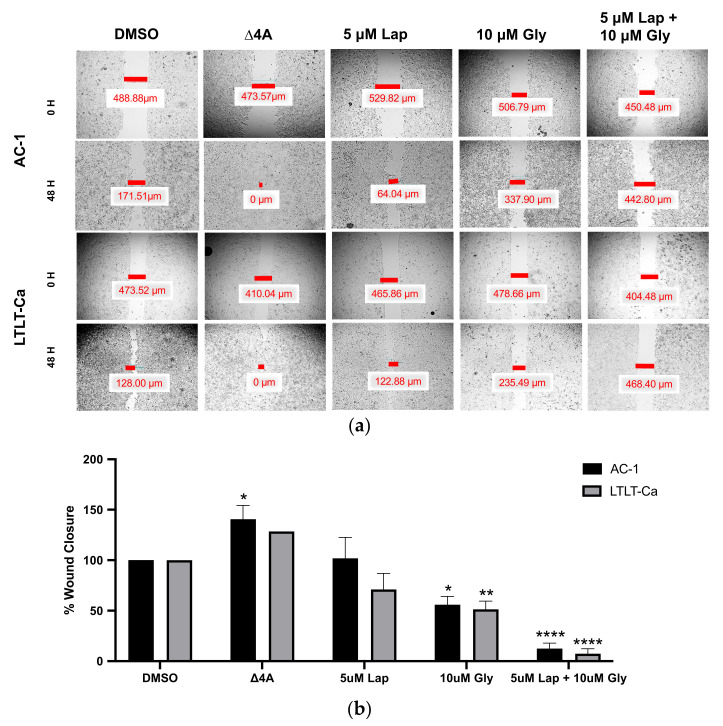
Lapatinib potentiates glyceollin-induced cell motility. Migration assays were performed using the AC-1 cells and the LTLT-Ca cells. Both cell lines were cultured in standard growth media and treated for 24 h with DMSO vehicle control, 25 nM androstenedione (Δ4A), 5 μM lapatinib, 10 μM glyceollin, and 5 μM lapatinib + 10 μM glyceollin as described in methodology. (**a**) Images depict the wound distance at 0 h and 48 h. (**b**) Graphical representation of the percentage of the wound closure. Two-way ANOVA analysis were conducted, and treatments were compared to the control. Results are expressed as the mean unit ± standard deviation of three independent experiments in triplicate. (**** *p* < 0.0001, ** *p* < 0.01, * *p* < 0.05) of three independent experiments in triplicate).

Previous reports demonstrate that as the letrozole-sensitive cells acquired resistance to letrozole, they became more migratory [23,25]. Therefore, we were interested in determining whether glyceollin ± lapatinib differentially inhibited cell motility between the two cell lines (Figure 3). Interestingly after 48 h of treatment, 5 µM lapatinib had no effect on the migration of both the AC-1 and LTLT-Ca cells. Alternatively, glyceollin prevented the migration in the AC-1 and LTLT-Ca cell lines by approximately 55% and 51%, respectively. The combination treatment exhibited inhibitory properties by preventing 88% wound closure in the AC-1 cells and 93% wound closure in the LTLT-Ca cells. Since combination therapy inhibited cell motility, we were interested in determining whether this observation was associated with alterations in mammosphere formation.

### 3.3. Glyceollin Inhibits Mammosphere Formation in Letrozole-Resistant Breast Cancer Cells

**Figure 4:**
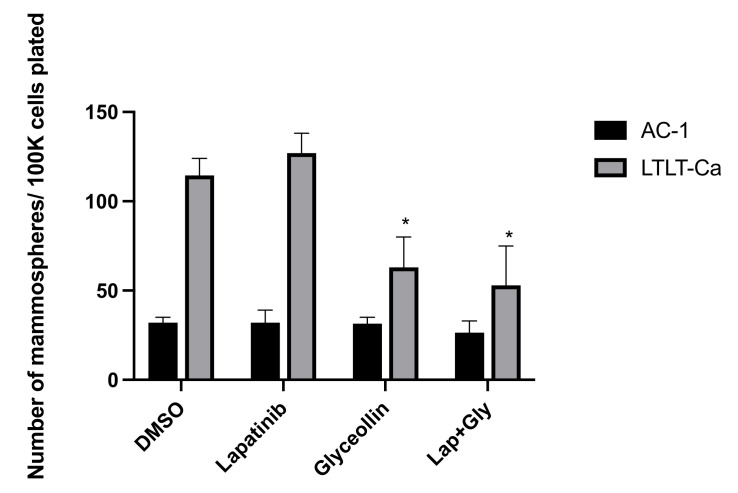
Glyceollin inhibits mammosphere formation in LTLT-Ca cells. Mammosphere formation assays were performed with AC-1 and LTLT-Ca cells. The total number of mammospheres was counted and plotted. * *p* < 0.05, where treatments were compared to vehicle control. Results are expressed as the mean unit ± standard deviation of 3 independent experiments in triplicate.

Since combination therapy inhibited cell motility, we were interested in determining whether this observation was associated with alterations in mammosphere formation. As previously demonstrated, the LTLT-Ca cells form mammospheres at a higher index than the AC-1 cells [25]. While single and combination treatment altered cell migration to a similar extent in both cell lines, neither lapatinib nor glyceollin had any effect on AC-1 mammosphere formation (Figure 4). While lapatinib did not alter LTLT-Ca mammosphere formation, glyceollin significantly decreased mammosphere formation, suggesting that glyceollin may play a role in targeting cancer stem cells.

### 3.4. Glyceollin and Lapatinib Induce S and G2/M Phase Cell-Cycle Arrest in Letrozole-Resistant Breast Cancer Cells

**Figure 5:**
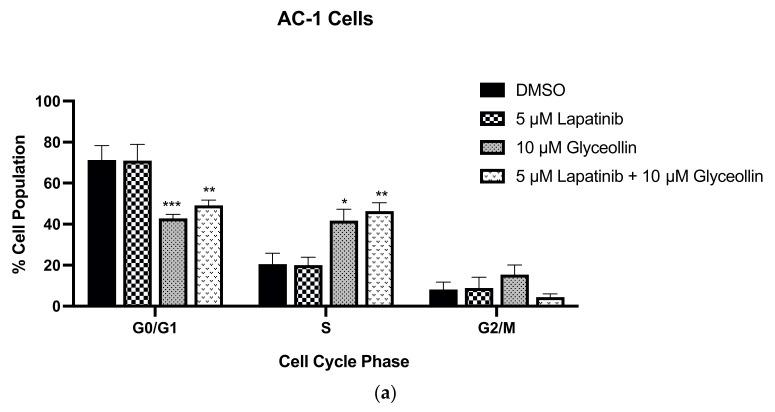

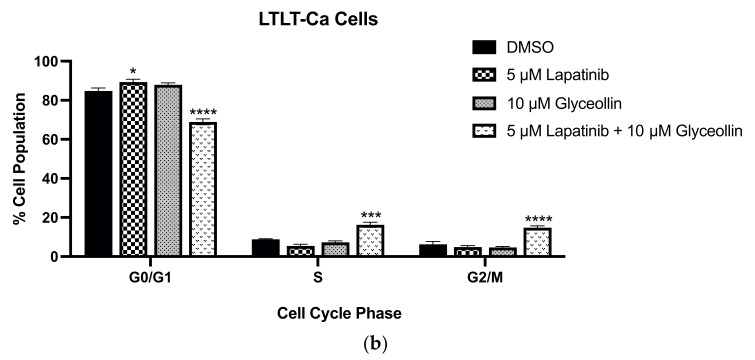
Glyceollin alone and in combination with lapatinib inhibits G1/S and G2/M cell cycle progression in LTLT-Ca cells. (**a**) The AC-1 and (**b**) the LTLT-Ca cells were assessed by flow cytometry for cell cycle progression after treatment with the vehicle control (DMSO), 5 μM lapatinib, 10 μM glyceollin, and 5 μM lapatinib + 10 μM glyceollin for 48 h. Cells were detached, washed, fixed, and stained with propidium iodide. Cell cycle analysis was determined as described in the methodology. Two-way ANOVA analysis were performed, and treatments were compared to the DMSO control. Results are expressed as a percentage of total cells ± SD (**** *p* < 0.0001, *** *p* < 0.001, ** *p* < 0.01, * *p* < 0.05) of three independent experiments in triplicate.

Cell-cycle dysregulation due to increased cytoplasmic cyclin E is partly responsible for AI resistance [27,28,29]. Additionally, glyceollin inhibits the growth of LNCaP prostate cancer cells through targeting G1/S phase cell transition [30], long-term estrogen-deprived (LTED) MCF-7 breast cancer cells [31], estrogen-dependent aromatase-inhibitor-resistant T47DaromLR cells [11], and estrogen-independent LTLT-Ca cells [23]. To understand whether the decrease in cell viability because of combination therapy was a consequence of cell cycle dysregulation, we performed flow cytometry studies. Compared to the vehicle treated AC-1 cells, glyceollin and glyceollin + lapatinib decreased the number of cells in G0/G1 while increasing the number of cells accumulating in S phase. When LTLT-Ca cells were treated with lapatinib, there was a slight increase in cells in the G0/G1 phase, while glyceollin decreased cells in this phase. Interestingly, glyceollin treatment caused accumulation in both S phase and G2/M phase (Figure 5), suggesting that alterations in cell cycle distribution may be responsible, in part, for the anti-proliferative properties of glyceollin + lapatinib in the LTLT-Ca cells but not the AC-1 cells. Resonant images of the results are reported in Supplemental Figure S1.

**Figure 6:**
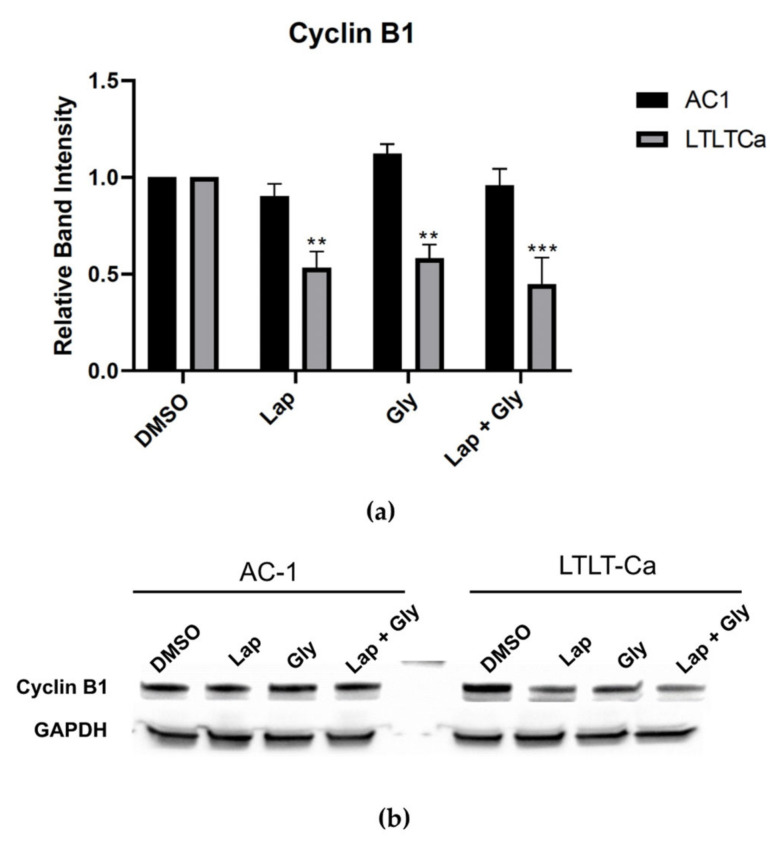
Lapatinib and glyceollin alters cell cycle progression mediating by cyclin B1. (**a**) The AC-1 and the LTLT-Ca cells were treated with the vehicle control (DMSO), 5 μM lapatinib, 10 μM glyceollin, and 5 μM lapatinib + 10 μM glyceollin (combo) for 24 h and assayed by immunoblot with antibodies directed against cyclin B1. GAPDH was measured as an internal loading control. Quantitative analysis representing the fold changes in cyclin B1 after normalizing to the GAPDH loading control. The AC-1 and LTLT-Ca cells were set to 100%. Two-way ANOVA analysis were performed, and treatments were compared to DMSO control. Results are expressed as the mean unit ± SD (*** *p* < 0.001, ** *p* < 0.01), and data are representative from one of at least three independent experiments. (**b**) Representative immunoblots of Cyclin B1 expression in the AC-1 and the LTLT-Ca cells. GAPDH was measured as an internal loading control.

Since combination therapy caused alterations in the distribution of the LTLT-Ca cells in various phases of the cell cycle, the cells were treated lapatinib ± glyceollin to ascertain the expression of key cell cycle regulators such as cyclin B1 (Figure 6) p21, p27, cdk1, and cdk2 (Supplemental Figure S2). Both cell lines were treated with glyceollin ± lapatinib, and there was no effect on protein expression in the AC-1 cells. However, glyceollin ± lapatinib treatment selectively decreased the expression of cyclin B1 in the LTLT-Ca cells without significantly altering the expression of the other proteins examined, suggesting that alterations in cell cycle distribution and proliferation observed in the LTLT-Ca cells are due in part to decreased cyclin B1.

## 4. Discussion

Previous studies demonstrated that AI-resistant cells are sensitive to the inhibitory properties of glyceollins [23,26]. Since it is unlikely that monotherapy (i.e., a single phytochemical) can exhibit a durable, long-term anti-cancer effect on refractory breast cancer cells, we chose to examine the effects of combining a phytochemical with lapatinib. Given the recent results from a Phase III clinical trial demonstrating dual HER2 blockade with lapatinib and trastuzumab in combination with an AI was associated with superior progression free survival compared to trastuzumab plus an AI in postmenopausal women with HER2-positive hormone-receptor-positive metastatic breast cancer [32], we were encouraged to study the combination of lapatinib plus glyceollin as AI-resistant cells overexpress HER2 and EGFR (i.e., which is the target of lapatinib). We were also interested in this therapeutic combination since our previous report demonstrated that glyceollin and lapatinib inhibited the proliferation of hormone-dependent AI-resistant T47D cells through induction of apoptosis [26]. Therefore, we chose to examine whether this combination would be effective in hormone-independent LTLT-Ca AI-resistant cells. When both the AI-sensitive and -resistant cells were examined for aromatase, EGFR, HER2, and ERα expression, we were surprised to find that although the ER levels in the LTLT-Ca cells were undetectable, compared to their AC-1 counterparts, the LTLT-Ca expressed higher levels of aromatase. A similar finding was also demonstrated in the T47DaromLR cells (i.e., aromatase overexpressing T47D cells treated with 10 μM letrozole for 75 weeks which acquired letrozole resistance) [33].

To determine whether the LTLT-Ca cells were estrogen-dependent, viability studies were conducted and after a 24 h treatment, androstenedione slightly induced the viability. This modest induction was not observed at later time points. This suggested that although the LTLT-Ca cells express higher levels of aromatase, it may be temporal and/or may not be fully functional. We also observed that the ER down regulator fulvestrant (ICI) inhibited the viability of the hormone-independent LTLT-Ca cells. While unexpected, previous reports demonstrate that fulvestrant significantly inhibited aromatase [34] and the growth of triple-negative breast cancer cells and tumors which was mediated by decreased ERβ levels [35]. While the LTLT-Ca cells are not considered triple-negative breast cancer cells, they may represent a transitory phenotype that is poised to progress towards a more aggressive and metastatic triple-negative phenotype.

Previous reports indicate that as the LTLT-Ca cells acquired resistance to letrozole, the morphology of the cells are altered to a mesenchymal-like phenotype that was reversed to an epithelial-like phenotype upon treatment with glyceollin [23]. The glyceollin-induced change in cell morphology was associated with decreased cell motility, decreased expression of the transcription factor ZEB1 and EGFR, and increased E-cadherin in vitro and in vivo, suggesting that glyceollin could potentially reverse EMT. While these results were promising, given that the LTLT-Ca cells overexpressed HER2 and EGFR and that glyceollin inhibited EGFR, we were prompted to test whether the combination of lapatinib (a dual HER2/EGFR inhibitor) and glyceollin could act synergistically to inhibit the proliferation and motility of the LTLT-Ca cells. Because the LTLT-Ca cells express higher levels of HER2 and EGFR than the AC-1 cells, we expected these cells to be more sensitive to the anti-proliferative properties of lapatinib and glyceollin and behave much like what was previously demonstrated with the AI-resistant T47DaromLR cells [26]. However, this was not the case, as the AC-1 cells and the LTLT-Ca cells responded similarly. One explanation for the similarities is that both cell lines express comparable levels of HER2 which is a target of lapatinib. Although both cell lines express varying levels of ERα, glyceollin as a single agent has been demonstrated to inhibit the proliferation of both estrogen-dependent and estrogen-independent breast cancer cell lines that were derived from the MCF-7 cell line [23,26] as well as inhibit tumor formation in triple negative breast cancer cell lines [24].

Interestingly, when wound healing assays were conducted, both cell lines responded similarly to glyceollin treatment, but lapatinib alone had no effect. When glyceollin was combined with lapatinib, there was a dramatic decrease in cell motility, suggesting that when the two agents are combined, they exhibit potentiation. Additionally, when mammosphere formation assays were conducted, not only did the LTLT-Ca cells form more mammospheres than AC-1 cells as previously observed [25], but glyceollin inhibited mammosphere formation, which may contribute to the anti-proliferative and anti-migratory effect of glyceollin. To confirm the precise mechanism responsible for the potentiation between glyceollin and lapatinib on LTLT-Ca cell migration, future studies will require careful identification of proteins involved in EMT, cell motility, and cell adhesion.

Cell cycle regulation plays a critical role in balancing normal cellular proliferation while ensuring that cells with DNA damage are not propagated to daughter cells. Various cell cycle regulators and check points are in place to maintain homeostasis and halt the progression of damaged cells. Since, previous reports by our lab indicated that glyceollin + lapatinib caused S phase arrest in the AI-sensitive T47Darom cells and S and G2/M cell cycle arrest in the AI-resistant T47DaromLR cells [11], we were interested in determining if combination treatment would follow a similar trend in the AC-1 and LTLT-Ca cell lines. As there were relatively few differences between the two cell lines with respect to how drug treatment impacted cell proliferation and migration, we expected that both the AC-1 and LTLT-Ca cell lines would exhibit a similar cell cycle progression profile. Interestingly, glyceollin and glyceollin + lapatinib decreased the number of AC-1 cells in G0/G1 causing accumulation in S phase, which could precede apoptosis, as demonstrated by others [36], while in the LTLT-Ca cells, combination therapy caused a decrease in G0/G1 and accumulation in S and G2/M that was accompanied by decreased cyclin B1. Other cell cycle mediators were examined but there was no change in cdk1, cdk2, p21, or p27. Downregulation of cyclin B1 is consistent with studies showing that this gene plays a role in inhibiting proliferation of several breast cancer cell lines [37]. This study also found that decreased cyclin B1 sensitized breast cancer cells to taxol. However, since cyclin B1 is the regulatory subunit of cdk1, which is essential for the transition from the G2 phase to mitosis, further studies are required to explain why decreased cyclin B1 was not associated with decreased cdk1.

## 5. Conclusions

While both AI-sensitive and AI-resistant cell lines are inhibited by the combination of glyceollin + lapatinib, future studies are required to distinguish cell line specific mechanism(s) responsible for the anti-proliferative and anti-migratory action, especially since cancer stem cells do not seem to contribute mechanistically to how AI-sensitive breast cancer cells respond to therapy. Interestingly, while both AI-resistant cell lines (LTLT-Ca and T47DaromLR) are sensitive to combination therapy, not all estrogen-dependent AI-sensitive cells respond favorably to combination therapy due to differences in the tumor microenvironment. AI-sensitive cells that are derived from MCF-7 cells (AC-1 cells) are sensitive to glyceollin + lapatinib, while AI-sensitive cells derived from T47D cells (T47Darom cells) are not. Taken together, understanding the individual mechanism(s) of inhibition among the AI-sensitive cell lines and between the AC-1 versus LTLT-Ca cells may represent a new avenue to develop strategies to target various subtypes of breast cancer.

We demonstrated that the combination of glyceollin + lapatinib selectively inhibits the proliferation of hormone independent LTLT-Ca cells through S and G2/M accumulation that is mediated by decreased cyclin B1. Thus, the observed association between the cyclin B1 dependent decreased proliferation and decreased cell cycle progression may suggest that cyclin B1 may be a potential target and/or biomarker that may be significant in AI resistance. Although our study demonstrates potentiation between lapatinib and glyceollin in AI-resistance, it remains unclear whether the pre-clincal impact of combination therapy on the cancer stem cell niche will translate to a durable clinical response in the metastatic setting. Finally, given that combination therapy caused decreased cyclin B1 expression in AI-resistant cells, this may represent a unique opportunity to expand the repertoire of cell cycle inhibitors that can be purposed for endocrine resistant HER2+ breast cancer.

## Data Availability

The data presented in this study are available in Supplemental Data Figures S1 and S2.

## Supplementary Materials

The following supporting information can be downloaded at: https://www.mdpi.com/article/10.3390/nu15071632/s1, Figure S1: Glyceollin alone and in combination with lapatinib inhibits G1/S and G2/M cell cycle progression in LTLT-Ca cells Representative histogram of the flow cytometry profiles monitoring cell cycle progression as determined by the distribution of DNA content. Two-way ANOVA analysis were performed, and treatments were compared to the DMSO control. Results are expressed as a percentage of total cells ± SD of three independent experiments in triplicate; Figure S2: The Effect of Lapatinib and Glyceollin Treatment on cell cycle regulator expression. The AC-1 and the LTLT-Ca cells were treated with the vehicle control (DMSO), 5 μM lapatinib, 10 μM Glyceollin, and 5 μM lapatinib + 10 μM Glyceollin for 24 h and assayed by immunoblot with antibodies directed against CDK2, p21, p27, and CDK1. GAPDH and β actin were measured as internal loading controls.
