# Supplementary material for: Novel Therapeutic Combination Targets the Growth of Letrozole-Resistant Breast Cancer through Decreased Cyclin B1
Source: Nutrients. 2023 Mar 28;15(7):1632. doi: 10.3390/nu15071632 (PMC10097176; doi:10.3390/nu15071632)
Supplement: Supplementary file 1 [file nutrients-15-01632-s001.zip › nutrients-2289682-supplementary.pptx]

## Slide 1
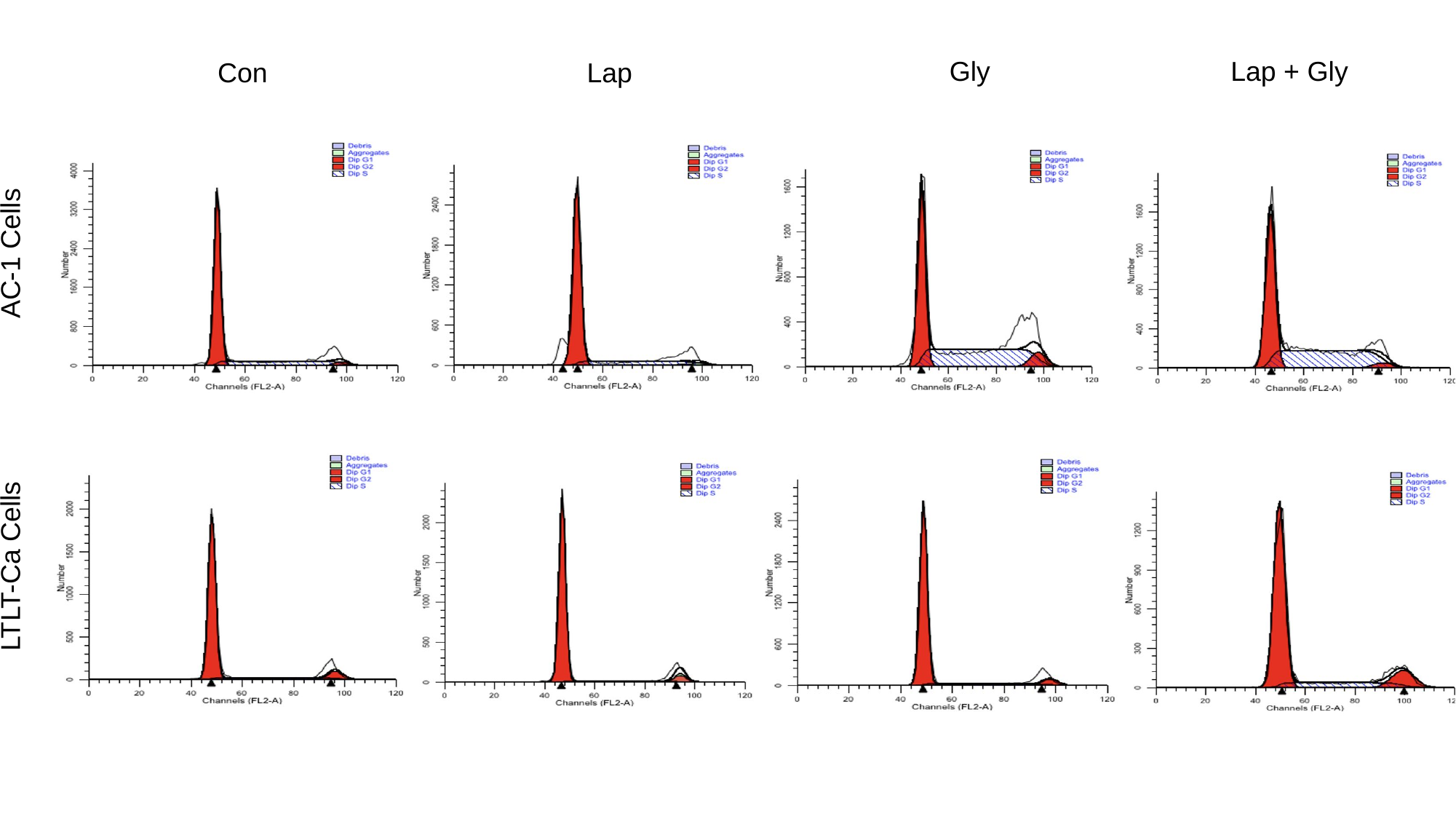

Gly
Lap + Gly
Con
Lap
AC-1 Cells
LTLT-Ca Cells

## Slide 2
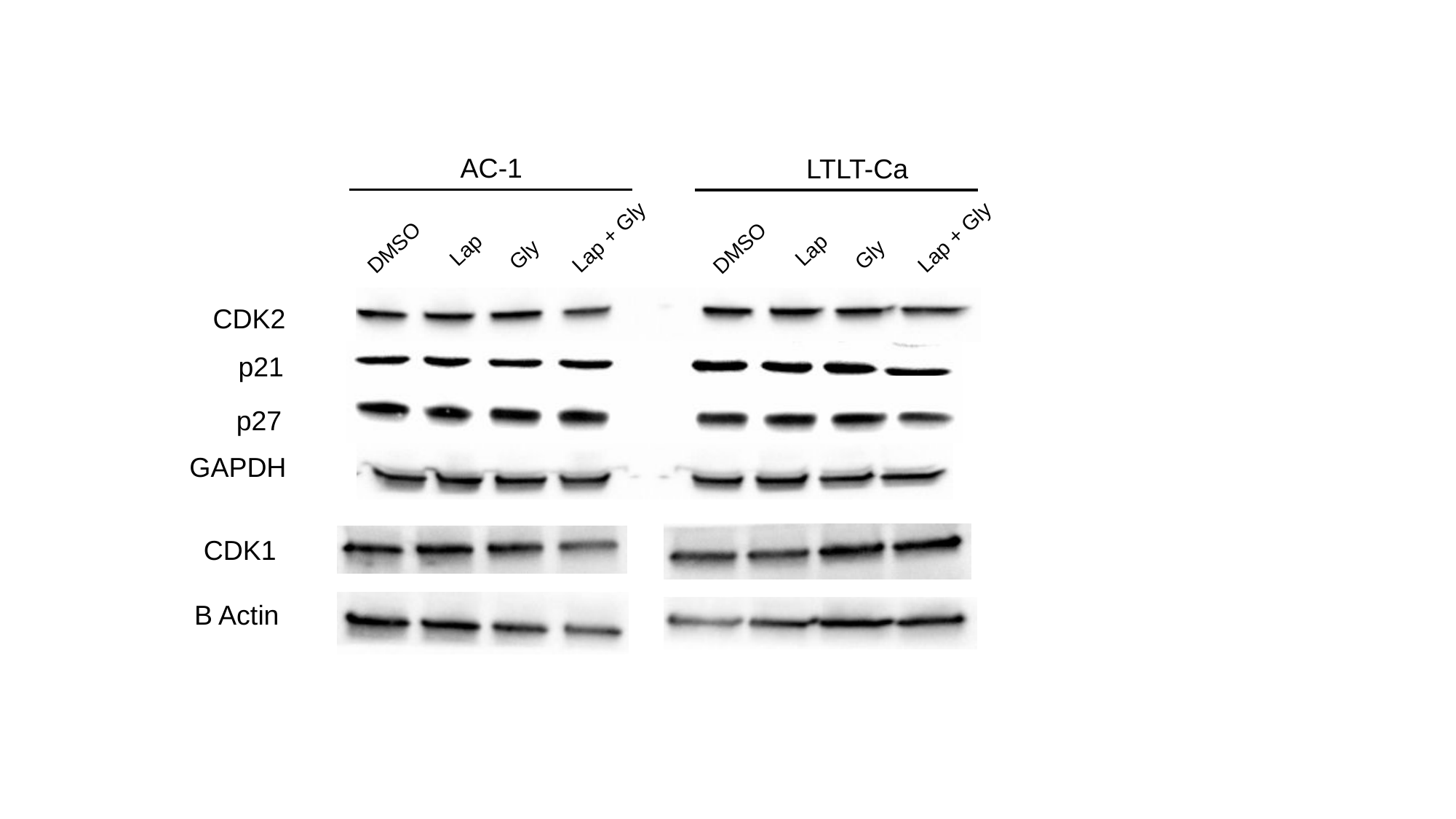

AC-1
LTLT-Ca
Lap + Gly
Lap + Gly
DMSO
DMSO
Lap
Lap
Gly
Gly
CDK2
p21
p27
GAPDH
CDK1
B Actin
